# A Magnetic Crawler System for Autonomous Long-Range Inspection and Maintenance on Large Structures

**DOI:** 10.3390/s22093235

**Published:** 2022-04-22

**Authors:** Georges Chahine, Pete Schroepfer, Othmane-Latif Ouabi, Cédric Pradalier

**Affiliations:** 1 College of Engineering, Georgia Institute of Technology, Atlanta, GA 30332, USA; pschroepfer6@gatech.edu; 2 IRL 2958 Georgia Tech-CNRS, 2 Rue Marconi, 57070 Metz, France; oouabi@georgiatech-metz.fr (O.-L.O.); cedricp@georgiatech-metz.fr (C.P.)

**Keywords:** robotics, sensor fusion, sensors, autonomous inspection, mobile robotics, ship hull inspection, autonomous, perception, ultrasonic

## Abstract

The inspection and maintenance of large-scale industrial structures are major challenges that require time-efficient and reliable solutions to ensure the healthy condition of structures during operation. Autonomous robots may provide a promising solution for this purpose. In particular, they could lead to faster and more reliable inspection and maintenance without direct intervention from human operators. In this paper, we present a custom magnetic crawler system, and sensor suit and sensing modalities to enable such robotic operation. We first describe a localization framework based on a mesh created from a point cloud coupled with Inertial Measurement Unit (IMU) and Ultra-Wide Band (UWB) readings. Next, we introduce a mapping framework that relies on a 3D laser, and explicitly state how autonomous navigation and obstacle avoidance can be developed. Lastly, we present how ultrasonic guided waves (UGWs) are integrated into the system to provide accurate robot localization and structural feature mapping by relying on acoustic reflections in combination with the other systems. It is envisioned that long-range inspection capabilities that are not yet available in current industrial mobile platforms could emerge from the designed robotic system.

## 1. Introduction

The marine industry is an essential element of economical activity. It is estimated that, every day, thousands of cargo vessels travel the seas for the shipping of goods. However, outer ship hulls deteriorate over time due to their operational conditions, for example, maritime environments can favor the emergence of corrosion patches in metallic ship structures and the formation of biofouling on the surface. In other industrial sectors such as the petrochemical industry, large structures such as storage tanks are also necessary and deteriorate over time due to fatigue, corrosion, creed, and other factors. Hence, the inspection and maintenance of large-scale structures are critical to ensure their healthy state, so that the risk of catastrophic failures can be mitigated.

Standard inspection and maintenance methods are time-consuming. Indeed, outer-hull service, inspection, and maintenance are mostly conducted at a dry dock, either manually or with a remote automated system. In this condition, complete hull thickness measurements are achieved by discrete sampling, but this accounts for 5–8 days of work. This may also be achieved in areas that are difficult to access or that present risks for inspection when carried out by a human operator. Overall, the mentioned methods are time-inefficient and usually have serious financial impact to the owners.

In this work, the integration of ultrasonic guided waves (UGWs) is presented. On metal plates, UGWs are generated by applying piezoelectric transducers in contact with the surface. These waves propagate radially around the emitter through the plate material and potentially over large distances. When encountering structural features (such as plate edges, stiffeners, weld joints), these waves are reflected, and the transducer collects ultrasonic echoes. In this setup, the resulting acoustic data carry essential information on source position and structure geometry. Hence, their integration into a mobile robotic system may lead to accurate robot positioning on the structure in combination with other sensors. Furthermore, as these waves are sensitive to thickness-altering flaws (such as corrosion patches), they can be used for inspection purposes to enable the long-term objective of long-range defect detection, which has not yet been established for a mobile system.

### Related Work

Autonomous robotic systems have not yet been demonstrated enough to convince owners and end users, whereas they could reduce inspection and maintenance costs while increasing operation efficiency. By bridging the gap between the current and desired capabilities of ship inspection and service robots, the recent development of adaptable autonomous robotic solutions can be used to detect corrosion patches and/or cleaning the surface of the outer hulls and storage tanks of ships. Increasing regulatory constraints, the desire to reduce inspection costs and human risk, and recent pandemic outbreaks have driven research in the field of autonomous inspection. The state of the art in autonomous robotic inspection contains sparse yet interesting venues for storage tank and ship inspection. For instance, the authors in [1] provided an autonomous approach to inspecting ship hulls, yet the approach was limited to weld lines and does not offer a global approach for other parts of the surface.

Other comprehensive approaches include [2], where the authors presented a magnetic autonomous robot for ship-hull inspection. The approach, however, does not support mapping capabilities, which hinders human monitoring. The proposed method seems especially focused on the mechanical design of the robot and does not sufficiently address the autonomous part. Additionally, the authors’ experiments were limited to lab testing, and unlike our approach, did not address obstacle detection or avoidance.

The literature discussing autonomous ship-hull inspection does not often address above-water robots. Nevertheless, a few contributions, such as [3,4,5] discussed the autonomous inspection of underwater parts of ship hulls. In addition to above-water robots, this project also included the development of autonomous underwater vehicles (AUVs) for ship-hull inspection and unmanned aerial vehicles (UAVs) [6]. However, this paper does not discuss the use of AUVs and UAVs (more information: https://www.bugwright2.eu/project/ (accessed on 7 March 2022)).

UGWs are usually deployed on sensor networks that are permanently attached to the structure for structural health monitoring (SHM) [7,8,9]. In this setup, however, only a fixed and restricted area can be monitored. The use of guided waves on a mobile robotic platform is emerging research topic in the literature. Recent works focus on the use of directional guided waves for mapping structural features with a mobile unit [10,11]. Omnidirectional guided waves were also considered for mapping defects with a mobile unit [12]. However, it is still a challenge for various reasons, namely, the complexity of the physics of wave propagation, which is multimodal and dispersive [7]. This means that propagation velocity depends on wave frequency. The interpretation of multiecho data in noisy environments (and in imperfect robot localization conditions) is also a particularly difficult challenge. However, recent advances were undertaken to enable the use of omnidirectional UGWs for acoustic localization and mapping purposes. In [13,14], the geometry of an isolated metal plate and the positions of a mobile colocated emitter/receiver pair of piezoelectric transducers were jointly estimated using UGWs echoes and a FastSLAM approach [15]. These works show the feasibility of embedding UGWs onto a robot. Still, experiments were conducted by manually moving the sensors without using a real robotic platform.

In this paper, we describe research on an above-water magnetic crawler prototype for the long-range inspection of storage tanks and ship hulls. This work is unique in both applications and implemented solutions for pose estimation, texture generation, and defect detection using guided waves. We also present an approach for the autonomous inspection of large structures. With a mature mechanical design, we focus on the autonomous parts of the system, namely, pose estimation (Section 3.1), obstacle detection (Section 4.1.2), mapping (Section 4.1.1), and defect detection (Section 3.2).

## 2. Experimental Platform: Altiscan Magnetic Crawler

The Altiscan magnetic crawler is a differentially driven robot equipped with magnetic wheels. The robot, manufactured by ROBOPLANET, is capable of driving on vertical surfaces, such as storage tanks and ship hulls. The crawler is also equipped with the following sensors:

- off-the-shelf 6-DOF IMU;
- off-the-shelf UWB receiver;
- off-the-shelf RGB camera;
- 3D Lidar: Livox Mid-70;
- embedded computer: Axiomtek CAPA310;
- rotary wheel optical encoders;
- contact V103-RM U8403008 piezoelectric transducer.

**Figure 1:**
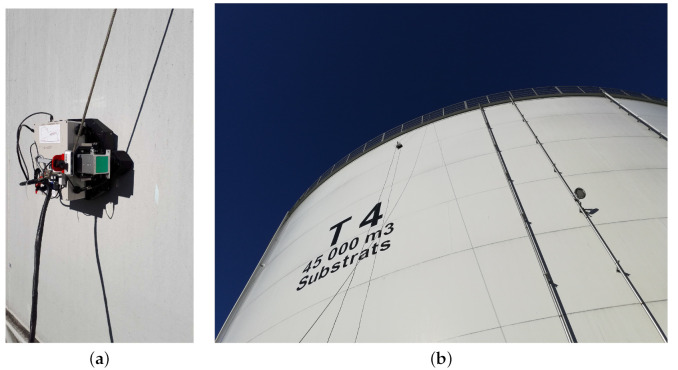
First prototype of magnetic crawler robot during a testing session near Bazancourt in France. (**a**) Close-up of magnetic crawler; (**b**) magnetic crawler on top of 20 m high storage tank.

Power, water, and data channels are bundled into a single cord connected to the crawler and the respective inputs. Water supply is essential to ensure adequate surface contact between the piezoelectric transducer and the metal surface. The crawler can be operated in manual and autonomous modes. Lastly, a picture of the magnetic crawler is shown in Figure 1.

## 3. Data Analysis and Interpretation

In this section, we present the scientific methodology adopted by the project towards the autonomous inspection of large structures. To that end, this section is divided into three parts: pose estimation, mapping and obstacle detection, and ultrasonic data processing.

### 3.1. Pose Estimation

A probabilistic algorithm for pose estimation is often necessary to create accurate localization. Currently, probabilistic algorithms such as the extended Kalman Filter (EKF), unscented Kalman Filter (UKF) and particle filter (PF) provide the best means of performing pose estimation for nonlinear systems with noisy measurements [16]. More specifically, the PF provides high accuracy with a nonlinear system while also providing multimodal pose distribution capable of handling non-Gaussian sensor noise [17,18,19].

#### 3.1.1. Particle Filter

In our case, the particle filter is an optimal localization algorithm given the dynamics of the crawler, coupled with the sensor package used for localization. As mentioned above, the crawler uses a differential drive, and the kinematic motion model for a differential drive is nonlinear. By using a PF that handles nonlinear motion models well and does not require linearization, the PF is a good choice for this type of motion. In terms of sensors, the crawler has only encoder readings (odometry), an IMU, and UWB measurements that it can use for localization. Range measurements such as UWB can lead to multimodal distribution when there are insufficient range measurements or high levels of noise, and often exhibit non-Gaussian noise due to time-of-flight measurements [17,19]. As the PF, unlike the EKF or UKF, produces no Gaussian assumptions regarding noise or the probability density function of the position, it is the filter that best matches data given by the sensors.

#### 3.1.2. Particle-Filter Mesh Projection

The major drawback of the PF for real-time use, as it tends to be computationally intractable in high dimensions due to bottlenecks in the algorithm coupled with a required high density of particles. In the case of wheeled robots, one solution is to only provide a pose estimate in SE(2) with 3 DoF [20,21,22]. Indeed, this solution works reasonably well for wheeled robots operating on a flat surface such as a road or hallway. However, once the robots starts to move on less planarlike surfaces, the mapping between the estimated pose in SE(2) with 3 DoF and the real pose of the robot within the world is not possible without introducing a high degree of uncertainty or error. Thus, for robots such as the crawler, which generally dives in complex three-dimensional structures such as ships or storage containers, this method would not be optimal.

**Figure 2:**
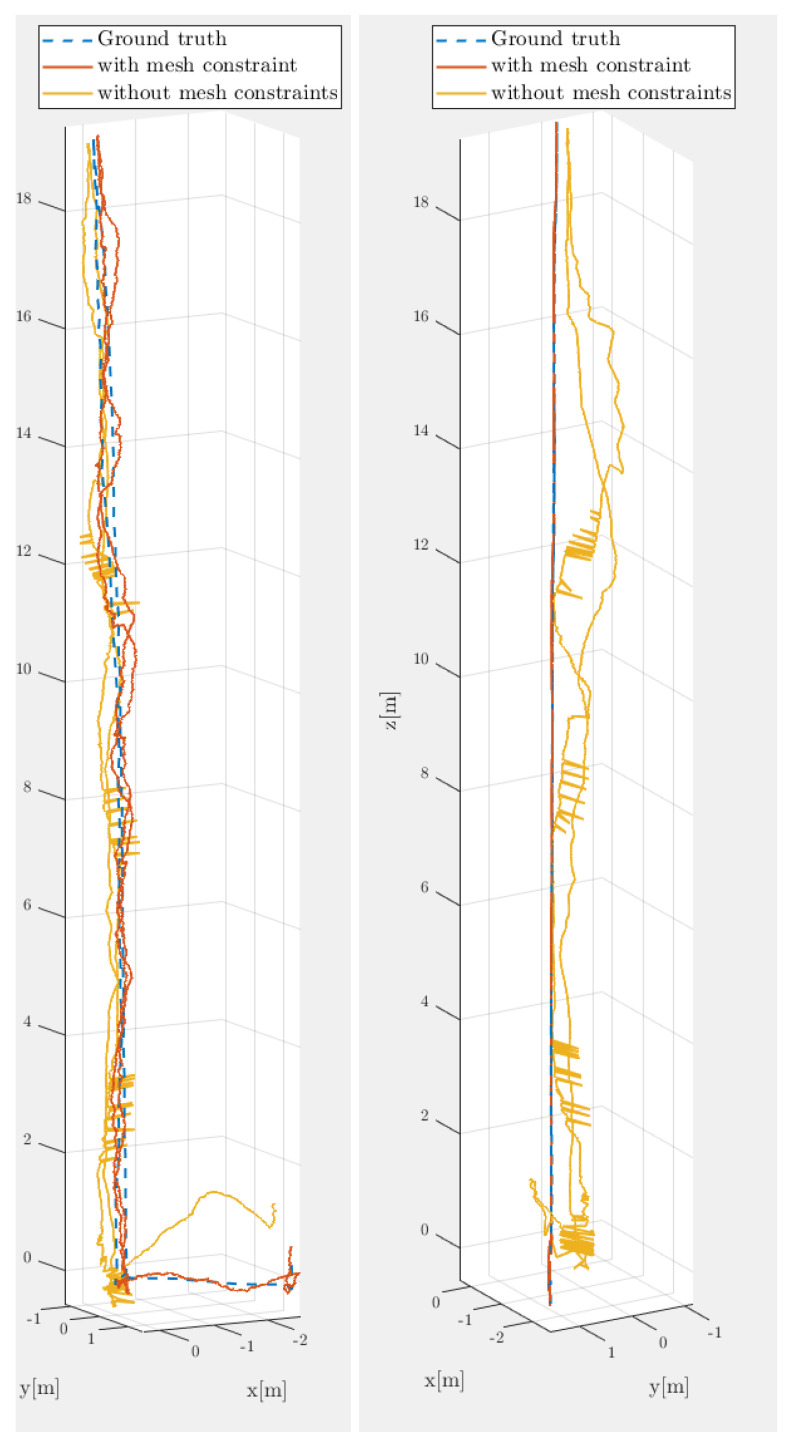
3D path comparison of 200 particle MCPF with a 20,000 SPF and ground truth ( front and side views).

One means of solving PF tractability problem is to reduce the required particles while maintaining a pose estimate in SE(3) with 6 DoF. Normally, this is not possible because, with 6 DoF, the set of possible poses after the robot moves is so large that a giant sample is needed to obtain an accurate estimate. However, if the set of possible poses after motion is applied (i.e., within the PF transition function) is reduced, lowering the particle count becomes possible without reducing the accuracy of the estimate. In this case, the crawler’s motion is actually constrained in such a way because it must move along a surface. These constraints reduce the effective workspace of the crawler, allowing for motion to be constrained to a surface (i.e., the set of possible poses in the world after motion is applied is constrained by the fact the crawler is moving on a surface, and the surface is known; this motion constraint can be seen in Figure 2, where the motion of the crawler can be seen as stuck to a surface when comparing mesh and nonmesh paths).

For the surface, manifold approximation in the form of a mesh is used during initialization and in the transitional function to reduce the workspace. To create this mesh, the surface on which the crawler moves is scanned to create a dense point cloud. On the basis of this point cloud, a mesh data structure is constructed that serves as an approximation of the smooth manifold on which the crawler moves. During the initialization and transitional functions, particles that are not on the surface of the manifold are then projected onto this manifold, ensuring that all particle positions are constrained to the mesh surface.

Once the position of the particles is constrained to the mesh surface, the orientation is further constrained so that it is consistent with the crawler moving on a surface. Here, the crawler (and by extension the particle) is attached to the surface of the manifold. To this end, the *x* axis in the robot frame is retained to preserve the equivalent of the yaw orientation of the crawler. The *z* axis of the crawler is then aligned with the normal vector of the manifold at the point where the crawler is located. The current orientation is then further constrained by aligning the *y* axis of the crawler to be perpendicular with the normal vector. In a sense, this can be seen as constraining the pitch and roll of the crawler to the mesh on a local plane while retaining the yaw.

**Figure 3:**
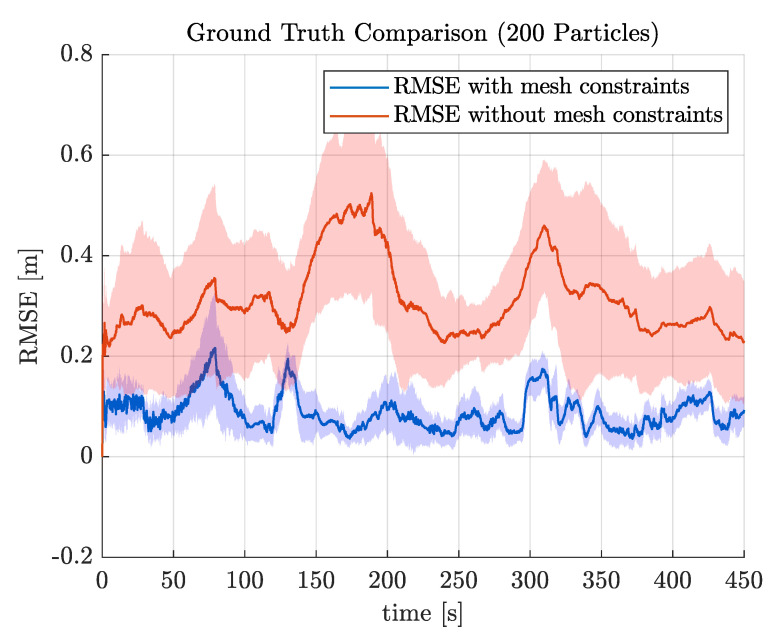
RMS translation error for 200 particles against ground truth obtained from a Leica MS60 Total Station. Shaded areas represent standard error deviation. The inclusion of mesh constraints dramatically improves position estimates and the standard deviation of the error.

By reducing the possible position and orientation of the robot, it is now possible to use a particle filter in the range of 200–500 particles while still maintaining a high level of accuracy (see Table 1 and Figure 3, both using the root mean square error (RMSE), defined below).

$$\mathrm{RMSE}=\sqrt{\frac{\sum_{t=1}^{T}{\left(\hat{y_{t}}-y_{t}\right)}^{2}}{T}}$$

### 3.2. Ultrasonic Guided Waves

**Figure 4:**
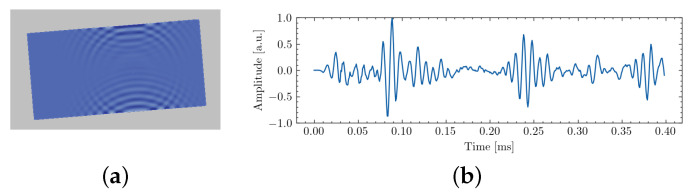
(**a**) Ultrasonic guided waves reflecting on the edges of a metal panel in a simulated environment. (**b**) Example of ultrasonic measurement acquired on an isolated metal panel in pseudo pulse-echo mode (i.e., with two nearly collocated transducers).

Autonomous robotic inspection on a large structure may be enabled by the use of ultrasonic measurements. Indeed, these waves may be leveraged to improve both localization and mapping capabilities by relying on acoustic echoes on the structural features (individual metal panel boundaries, stiffeners…), as illustrated by Figure 4. Furthermore, they can be used to enable long-range inspection, which has not yet been established for current robotic systems. In the following, we describe how UGWs are integrated in the current magnetic crawler and how they can be leveraged to achieve ultrasonic mapping.

#### 3.2.1. Integration of UGWs

**Figure 5:**
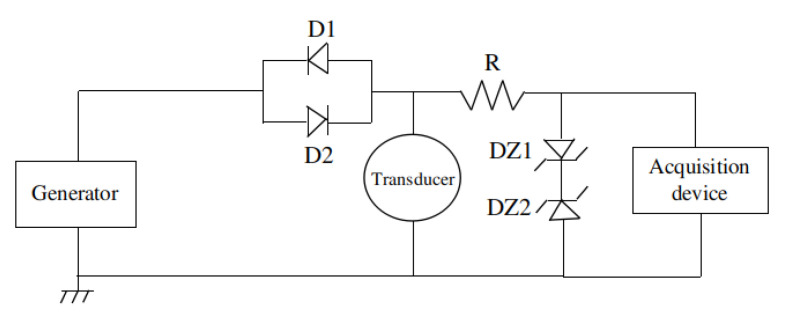
Schematic of designed electrical circuit to use a true pulse-echo setup. D1 and D2 are commutation diodes, DZ1 and DZ2 are Zener diodes, and R is a resistor.

In this part, we describe how UGWs were integrated within our magnetic crawler system. The robot is equipped with a single piezoelectric contact transducer placed in its head. This is in contact with the surface to simultaneously generate and receive UGWs propagating in the material. We used an electrical circuit, shown in Figure 5, to emit and receive UGWs using a single transducer in true pulse-echo mode. The principle was to protect the acquisition device during excitation at high amplitude. Otherwise, the acquisition device could be damaged. This was achieved by the use of Zener diodes that limited the voltage level at their ends while only inducing little deformation on low-amplitude signals (that contained the acoustic echoes). The robot was also equipped with a tether that carried a tube to continuously bring water at the interface between sensor and surface with an electrical water pump.

The sensor used for our prototype system was a contact V103-RM U8403008 piezoelectric transducer. This sensor was used as it is a common industrial sensor that was easily available. Although it can generate and receive UGWs, it is not an optimal choice, as it should be used for 1 MHz signals; for our application, lower frequencies are typically used.

#### 3.2.2. Ultrasonic Mapping via Beamforming

The chore of our methodology to achieve acoustic localization and mapping was beamforming [23]. This spatial filtering approach can focus, in the localization space, the energy contained in the measurements acquired over the robot trajectory. Hence, acoustic reflectors (such as individual metal plate boundaries) can be localized by retrieving local maxima on beamforming results. The principle of this strategy was successfully demonstrated in [14] to achieve acoustic SLAM. An example of plate geometry reconstruction using our magnetic crawler system is provided in the Section 4.

## 4. Field Experiment

### 4.1. Mapping and Obstacle Detection

In this section, we present our approach to the mapping problem while taking into account contextual constraints such as obstacles and the need to detect free space.

Maps are important to robots since they can be used for obstacle avoidance, path planning, and to constrain the attitude of the robotic system. Maps are also important to human operators looking for visual feedback from the robot’s perspective.

As previously discussed, the robot was equipped with an RGB camera, a 3D lidar, and an IMU. In addition, optical sensors capture wheel odometry. Further, as discussed in Section 3.1, an Extended Kalman Filter (EKF) [24] filter was used to fuse IMU and wheel odometry data. For the remainder of this article, the obtained pose is further referenced to as the “EKF pose”.

To compensate for uncertainties such as drift, pose correction is performed using a constrained version of Iterative Closest Point (ICP). The latter approach is further discussed in Section 4.1.3.

#### 4.1.1. Stop and Map

The majority of filtering techniques, such as the Kalman filter [25], introduce time delays between filter estimates and actual observations. In that sense, the generated estimate satisfied control requirements for autonomous driving. Nevertheless, mapping was more challenging. To solve this problem, a stop-and-map approach was implemented in the autonomous planner, i.e., the robot task manager.

**Figure 6:**
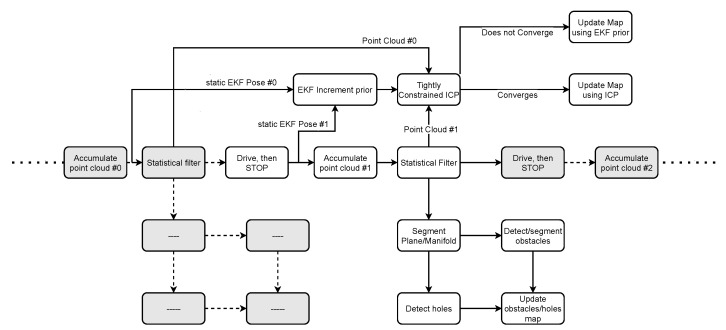
Flowchart of proposed mapper: grayed-out rectangles denote repeated behavior.

As shown in Figure 6, the proposed mapper is idle while the robot is moving and only captures data when the robot stops. Once static, the point cloud is accumulated, and the pose is captured.

**Figure 7:**
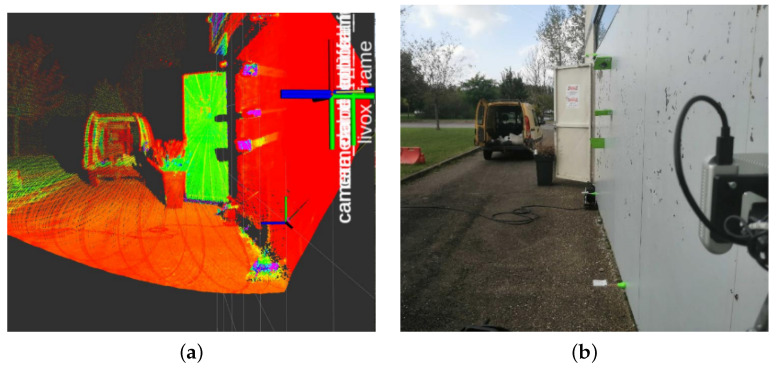
RGB vs. intensity map showing metal plates used to test the magnetic crawler robot in Metz. (**a**) Accumulated intensity point cloud taken from the Livox Mid-70; (**b**) RGB camera feed.

The latter approach thus does not suffer from data delays. The EKF pose is then captured around half a second after the robot stops. Minimal stop time was set to 3 s to allow for the on-board laser to accumulate sufficient points for data fitting. This is especially useful with 3D lidars equipped with a scanning unit, such as the Livox Mid-70. A sample accumulated cloud can be seen in Figure 7.

#### 4.1.2. Obstacle Detection

The accumulated point cloud was then voxelized and processed through RANSAC [26] by fitting a second-degree manifold [27]. The choice of a second-degree manifold is rooted in the application in which the mapper is used: ship hulls and storage tanks using autonomous robots for inspection are often significant in size. As a result, nonflat surfaces comprise a significant radius. The curvature is thus locally negligible, i.e., the surface around the current position of the robot can be represented as a plane. Nevertheless, a second-degree manifold better captures surface geometry at unique places with an important curvature, such as at the tip of the ship structure.

Lastly, RANSAC inliers denote free observable space that belongs to the detected manifold, and outliers denote positive obstacles such as protruding objects and negative obstacles such as holes.

#### 4.1.3. Pose Correction and ICP

ICP is an algorithm used to stitch overlapping point clouds. It works by iteratively finding the transformation that better aligns point cloud pairs. An ICP prior on the transformation to-be-found improves the chances of converging to a valid solution [28].

Odometric EKF still suffers from translational drift. To that end, ICP is used between accumulated point clouds to reduce drift between successive stops. Nevertheless, ICP does not always properly converge on featureless surfaces. To overcome this issue, a constrained version of ICP was implemented. The purpose of these constraints is to prevent ICP from reducing the quality of the estimated EKF pose when it does not properly converge. The list of constraints can be found in Table 2.

After running few ICP iterations and due to point cloud overlap, the density of points should be standardized for both the newly accumulated point cloud and the previous ICP map. To that end, a density filter is applied to both inputs. Although filter value depends on point cloud density, its true purpose is to have the same density (value) for both inputs. The full list of ICP parameters is listed in Table 3.

Lastly, the map pose is corrected according to Equation (1), where *C* is the ICP correction inferred by matching the current accumulated cloud to the previously accumulated point cloud, *P* is the current pose in the reference frame of the map, $P_{c_{prev}}$ is the previously captured EKF pose when the robot was still static, and $P_{c_{new}}$ is the most recently captured pose with the robot also being static.

(1) $$P_{new}=P_{prev}P_{c_{prev}}^{-1}P_{c_{new}}C$$

#### 4.1.4. From Point Clouds to Texture Maps

Up to this point, the proposed framework still lacked a high-level visual component to be used by the system operator for visual feedback, manual driving, and debugging a possible snag. So far, point clouds have proven to be versatile data containers, and they are the precursors to creating maps. Nevertheless, there is a need for a representation that is finite in space and intelligible for people who are not point cloud experts. To that end, a multilayer texture map was conceived.

The generated texture is a projection of the RGB image on the robot surface. In the latter context, we assumed that the ground was flat. Ground pixels are projected onto the camera frame for color extraction. For this, we used pinhole model $p=A\left[R|t\right]P_{g}$, where $P_{g}$ is a 3D ground point, [R|t] is the extrinsic matrix that provides the geometric connection between lidar and camera frames, and *A* is the camera intrinsic matrix obtained by checkerboard calibration. Lastly, the colors of ground points $P_{g}$ are inferred by copying the colors of the nearest pixel after projection, i.e., those of p(u,v,1).

We projected the RGB image onto the ground surrounding the robot. What followed was the fusion of relevant semantic information, such as free spaces and obstacles, extracted from point cloud data. As such, pixels not seen by the lidar i.e., unobservable space, are marked in black, pixels belonging to obstacles are marked in red, and free space keeps the original RGB colors. The texture map comprises 3 layers:

- A bottom layer consisting of a dynamically updated projection of the ground portion of the image drawn at the estimated pose.
- A middle layer that overwrites the bottom layer using a clean representation, updated every time the robot stops.
- A top layer consisting of metadata such as grid resolution.

### 4.2. Ultrasonic Mapping

**Figure 8:**
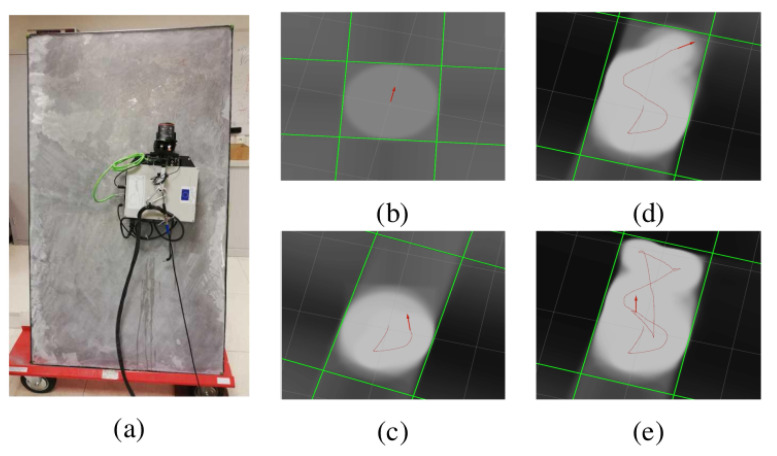
Plate geometry mapping with our robotic platform relying on UGWs. (**a**) Experimental setup. (**b**–**e**) Mapping results at different steps along robot trajectory. Red arrow, robot pose; red line, trajectory.

At the time of the writing, our prototype setup for emission and reception of UGWs was not yet fully established for successful operation on a large structure such as a storage tank. Still, we obtained experimental results with our system in a laboratory environment to only map the boundaries and the inner surface of an isolated 1700 × 1000 × 6 mm steel plate that was placed nearly vertically. A picture of the experimental setup is provided in Figure 8a. In the experiment, IMU readings were integrated into the robot trajectory estimation for accurate robot heading. However, UGW measurements were not used for robot localization.

Obtained results using the beamforming method presented in [29] are provided in Figure 8b–e at different steps of the robot transect resulting from manual driving. Results show that acoustic mapping using UGWs and our magnetic crawler system is feasible, as the plate dimensions and plate orientation with respect to the robot are recovered with sufficient precision. Detailed results are available in [29]. Overall, the experiment demonstrated the feasibility of ultrasonic mapping using guided waves and our magnetic crawler system. In future work, the combination of UGW measurements with other sensors and their deployment in more realistic environments should be investigated to achieve acoustic SLAM and eventually long-range defect detection.

### 4.3. Autonomous Navigation

The autonomous navigation system on the crawler consists of a mission system and a set of commands or tasks that can be used by the mission system. The mission system allows for the user to plan and create a mission in advance, consisting of a set of commands or tasks affecting the crawler’s behavior. For example, the crawler can schedule a task to continually monitor for objects while simultaneously performing a vertical transect for a set distance or until it reaches a set height.

In addition to more basic commands, such as performing vertical and horizontal transects, and rotations, the crawler can also utilize a mesh for control. In this case, the crawler gives a position on or near the mesh, generates a path to this position, and follows the trajectory to the position.

## 5. Infield Intervention

In this section, we show how the proposed system performed during infield interventions. To validate our system, a Leica MS-60 total station was used to track the real-time position of the crawler robot. Using NTP and PTP protocols, time synchronization was performed among on-board sensors, the on-board computer on the crawler, the total station, and the operator’s computer.

The experimental environment consisted of the large metallic storage tanks shown in Figure 1b. During the experiments, the robot was given the task of autonomously driving on the structure. Further, the on-board lidar was used to detect nearby obstacles. During the entire process, the robot captured ultrasonic measurements while mapping the structure in real time.

### 5.1. Pose and Autonomy Evaluation

For the mission on the metallic tank, the crawler was given the goal of performing a vertical transect to the top of the tank, returning to the bottom, rotating, and then performing a horizontal transect (see Section 2). The particle filter ran in real time on the crawler’s embedded computer with 200 particles. With 200 particles, the crawler was also able to use this localization for control due to the high accuracy of the particle filter (see Section 1).

In addition to the standard controls, we were also able to test the mesh-based control. In this case, the crawler was able to successfully create a path on the mesh data structure and use that path to navigate to the given location on the real surface.

### 5.2. Mapping and Texture Generation

**Figure 9:**
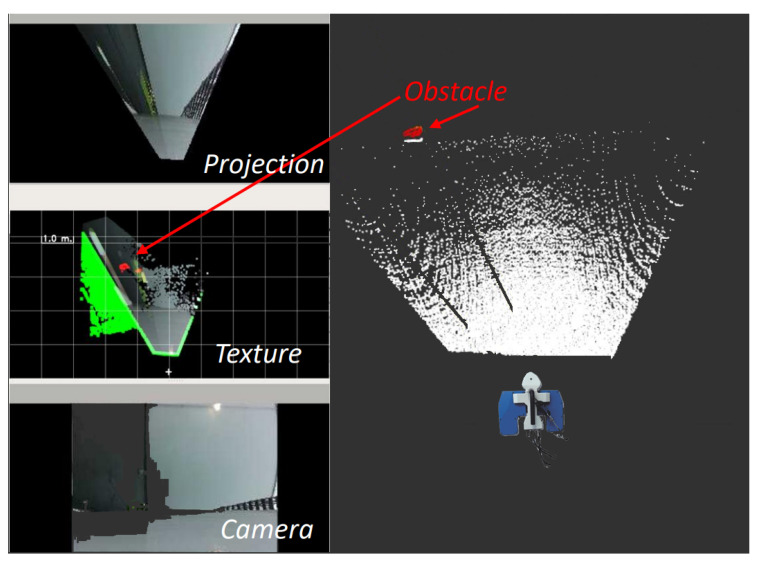
Accumulated point cloud with obstacle detected. Texture map shows free space in real world colors, the same obstacle detected in the point cloud (red), and previously observed free space (green).

As shown in Figure 9, the mapping algorithm provided semantic information that could be interpreted by both humans and computers. As expected, ICP did not always converge, but it improved the result when it did. When ICP did not converge, the fused pose was used instead.

## 6. Conclusions

This paper showed the potential afforded by autonomous inspection vehicle systems. By using the state of the art in localization, mapping, and the processing of ultrasonic guided waves, we showed how we can create an autonomous systems capable of navigating on its own while providing qualitative visual and quantitative feedback through the analysis of map and ultrasonic data. The development of such techniques is crucial to lowering costs associated with storage tank and ship inspections, and to decrease the already significant human risk. In this work, we showed how to generate and improve pose estimates, for instance, by using the mesh of the structure to constrain the pose. Semantic texture maps were also generated for navigation and obstacle avoidance. Lastly, we integrated ultrasonic measurements in our system to localize the boundaries of a metal panel using ultrasonic echoes.

Future work consists of improving the robot autonomy, for instance, by integrating data reported from the Unmanned Aerial Vehicle (UAV)s, Autonomous Underwater Vehicle (AUV)s, and the above-water crawler. The latter integration is needed to achieve a comprehensive understanding of structural integrity and for the precise localization of defects.

## Figures and Tables

**Table 1:** Average RMSE comparison.

Particles	Mesh	NoMesh
200	0.0856	0.3694
500	0.0780	0.3389
20,000	-	0.2532

**Table 2:** List of ICP constraints.

Constraint Type	Value
2D Constraint	ϕ=θ=Z=0
Maximal rotation norm	0.05 rad.
Maximal translation norm	0.35 m.
Minimal differential rotation error	0.01 rad.
Minimal differential translation error	0.01 rad.

**Table 3:** List of ICP parameters used for pose correction.

Parameter Name	Mapper
Matcher	KD tree matcher
Matcher KNN size	15
Error minimizer size	Point to Plane
Max iterations	25
Octree grid filter	0.01
Maximal input point density	400,000
Maximal ICP map point density	400,000

## Abbreviations

The following abbreviations are used in this manuscript:

EKF	Extended Kalman filter
UGW	Ultrasonic guided wave
PF	Particle filter
ICP	Iterative closest point
SLAM	Simultaneous localization and mapping
GPS	Global positioning system
IMU	Inertial measurement unit
DoF	Degrees of freedom
SLERP	Spherical linear interpolation
RMS	Root mean square
CAD	Computer-aided design
UWB	Ultrawide band
MDPI	Multidisciplinary Digital Publishing Institute
